# Redefining successful primary PCI

**DOI:** 10.1093/ehjci/jey159

**Published:** 2018-11-21

**Authors:** Peter J McCartney, Colin Berry

**Affiliations:** 1 British Heart Foundation Glasgow Cardiovascular Research Centre, Institute of Cardiovascular and Medical Sciences, 126 University Place, University of Glasgow, Glasgow, UK; 2 West of Scotland Heart and Lung Centre, Golden Jubilee National Hospital, Glasgow, UK

**This editorial refers to ‘Intramyocardial hemorrhage and prognosis after ST-elevation myocardial infarction’ by S.J. Reinstadler *et al.*, pp. 138–146.**

Restoration of coronary blood flow with primary percutaneous coronary intervention (PCI) is an effective treatment for ST-segment elevation myocardial infarction (STEMI), and primary PCI is the evidence-based standard of care for STEMI patients presenting within 12 h of symptom onset.^1^ On the other hand, restoration of epicardial blood flow results in reperfusion injury with failed myocardial perfusion in approximately 50% of patients,^2^ typically in the context of a successful primary PCI procedure. Procedure success defined as normal antegrade coronary blood flow is achieved in >95% of patients during daily practice.^3^^,^^4^

Failed myocardial reperfusion is a complex, heterogeneous microvascular problem. Several mechanisms have been implicated, including intra-vascular problems, such as distal embolization of thrombus/atheroma and extravascular problems, such as extrinsic microvascular compression due to intracellular (e.g. cardiomyocyte) and extracellular oedema.^5^ Taken together, these pathologies manifest clinically as microvascular obstruction (MVO).

Endothelial cells may be more resistant to ischaemia than the cardiac myocte,^6^ but eventually sustained ischaemia leads to endothelial dysfunction. Endothelial damage leads to impaired capillary integrity, tissue oedema and extravasation of red bloods cells into the extracellular space. Multiple studies have shown that MVO and intramyocardial haemorrhage (IMH) are closely related. In general, IMH does not occur in the absence of MVO but, on the other hand, MVO commonly occurs in the absence of IMH.^2^ The dynamic nature of MVO supports the concept that it may be reversible and thus a therapeutic target. On the other hand, IMH is a downstream pathological consequence of irreversible microvascular damage.^7^ The occurrence of IMH therefore represents failed myocardial reperfusion, and a failure of the therapeutic strategy.

MVO is a predictor of poor outcome independent of infarct size.^8^ Patients with MVO are more likely to develop heart failure post-MI with increased mortality. The prognostic significance of IMH has been the subject of much debate. In a study of 286 patients presenting with acute STEMI, we found that myocardial haemorrhage (identified by T2* imaging) was more closely associated with all-cause death and heart failure during 2.3 years follow-up when compared with MVO alone.^2^

The pathophysiological mechanisms linking IMH with worse outcomes independent of infarct size and MVO are incompletely understood. Key to this may be persistent local tissue inflammation within the infarct core in response to persistence of haemoglobin breakdown products and accumulation of deoxidized iron residues and tissue fibrosis. These pathologies prevent the natural healing process that otherwise would normally occur in reperfused myocardium in the absence of MVO and IMH. Cigarette smoking and a history of hypertension are risk factors for IMH. Carberry *et al.* demonstrated that persistent iron affected one in five patients who survived through to 6 months post-STEMI and was associated with adverse LV remodelling, worsening ejection fractions at 6 months. Systemic inflammation at baseline, reflected by the neutrophil count, was a univariable associate of persistent iron at 6 months, and presenting heart rate and a history of hypertension were multivariable associates.^9^ Additionally, iron deposition within the infarcted myocardium may have deleterious effects on the electrical stability of the heart and so may increase the likelihood of compromising ventricular arrhythmias and sudden cardiac death post-MI.^10^

Cardiovascular magnetic resonance (CMR) is the only method available to clinicians to detect this problem *in vivo*. T2* imaging is generally accepted as the reference method for the assessment of IMH in STEMI patients,^11^ and T2* imaging is increasingly available as an option in standard CMR protocols. Blood degradation products such as deoxyhaemoglobin exert a paramagnetic effect, reducing the T2* signal, represented by hypointense areas within the infarct core. Still, local signal loss due to artefact can complicate the imaging read-out, especially if supporting features such as reduced wall motion and infarction are absent.

Reinstadler *et al.*^12^ provide additional evidence for the clinical importance of IMH characterized by T2* imaging post-STEMI. They conducted a prospective multicentre study of 264 STEMI patients presenting within 12 h of symptom onset undergoing primary PCI. The primary endpoint was a composite of death, reinfarction, and new congestive heart failure at 12 months. Sixty patients had IMH, of these, 9 (15%) had major adverse cardiac events (MACE), whereas only 10 (4.9%) patients without IMH experienced a MACE. IMH was independently associated with MACE, and IMH increased the prognostic value of a model which included MVO. This study adds to the previous work by Carrick *et al.*,^2^ reaffirming IMH as a determinative pathological complication post-STEMI.

The study by Reinstadler *et al.*^12^ did have some limitations. The number of MACE events (*n* = 19) was modest and in isolation the results have qualified significance. On the other hand, these data are consistent with other studies.^2^^,^^13^ Reinstadler *et al.* highlighted five patients with IMH but no MVO which is not consistent with previous studies. In a serial imaging time course sub-study of 30 patients, MVO had resolved by day 10 in 44% of affected patients, with persistence of IMH in 25% of these.^2^ One potential explanation is the differing time-course of these pathologies with resolution of MVO in the presence of persistent IMH not disclosed by imaging at a single time-point up to 7 days. Imaging artefact may also be relevant. A small amount of MVO may not be visible within a zone of late gadolinium enhancement imaging and T2* artefact occurs at a tissue–air interface such as the infero-lateral wall of the left ventricle which may be mistaken for haemorrhage.

Therefore, IMH represents a target for preventive therapy, however, aside from timely reperfusion, there are no specific treatments for this problem. Favourable results in preclinical studies have not translated when assessed in patients.^14^ Randomized controlled clinical trials of novel therapeutic approaches designed to reduce the extent and severity of infarction, including novel cardioprotective interventions such as intra-venous beta-blocker therapy before reperfusion (EARLY-BAMI),^15^ intravenous inhibitors of mitochondria-mediated reperfusion injury, i.e. cyclosporine (CIRCUS),^16^ TRO40303 (MITOCARE),^17^ post-ischaemic conditioning (DANAMI-3 iPOST),^18^ and deferred stenting (DANAMI-3-DEFER),^19^ have not proven beneficial and intra-coronary vasodilator therapy with adenosine was actually harmful (REFLO-STEMI).^20^ A *post hoc* analysis of the Phase 2 METOCARD-CNIC trial^21^ indicated intravenous beta-blocker therapy might reduce the risk of MVO through inhibition of neutrophil recruitment and platelet activation. MVO is now identified as a therapeutic target in practice guidelines,^22^ but the gaps in evidence on the causes and treatment of IMH highlight the need for more research.

We have recently conducted a Phase 2 clinical trial of low dose adjunctive intracoronary fibrinolysis with alteplase in reperfused STEMI (ClinicalTrials.gov: NCT02257294). The clinical strategy involved identifying patients in whom initial coronary angiography identified occluded infarct-related artery and/or with a high thrombus burden. These characteristics place the participants at an increased risk of MVO. By targeting thrombus within the infarct-related artery and microcirculation with fibrinolytic therapy the aim was to restore microvascular blood flow at the earliest point after coronary reperfusion. On the other hand, the intervention has the potential to promote bleeding within the infarct zone and systemically. The risk of IMH was purposefully mitigated by selecting patients presenting with a comparatively short ischaemic time (<6 h) in whom radial artery access was used. The overall objective was to conduct a safety, efficacy and mechanisms evaluation within the context of a trial that was sufficiently large to give definitive results.

In conclusion, given that failed myocardial reperfusion occurs in one in every two patients undergoing primary PCI for acute STEMI, and IMH is an independent driver of prognosis, can primary PCI really be considered successful when these eventualities routinely occur? We think not. We propose that successful primary PCI is redefined as restoration of normal coronary blood flow in the absence of MVO and IMH. However, until specific evidence-based treatments for MVO and IMH become available, routine imaging with CMR to assess for these pathologies cannot be justified on economic grounds, and clinicians should follow optimal guideline-directed management for their post-MI patients.

## Funding

This work was supported by the British Heart Foundation (BHF) Centre of Research Excellence Award (RE/13/5/30177) and a research grant from the National Institute of Health Research Efficacy and Mechanism Evaluation Programme (12/170/45).

**Conflict of interest:** The University of Glasgow holds a research agreement with Siemens Healthcare.
